# Dietary patterns of 6–24‐month‐old children are associated with nutrient content and quality of the diet

**DOI:** 10.1111/mcn.12901

**Published:** 2019-11-14

**Authors:** Mieke Faber, Marinel Rothman, Ria Laubscher, Cornelius M. Smuts

**Affiliations:** ^1^ Non‐Communicable Diseases Research Unit South African Medical Research Council Cape Town South Africa; ^2^ Centre of Excellence for Nutrition (CEN) North‐West University Potchefstroom South Africa; ^3^ Department of Consumer and Food Sciences University of Pretoria Pretoria South Africa; ^4^ Biostatistics Unit South African Medical Research Council Cape Town South Africa

## Abstract

We determined the associations of dietary patterns with energy/nutrient intakes and diet quality. Previously collected single 24‐hr dietary recalls for children aged 6–11 months (*n* = 1,585), 12–17 months (*n* = 1,131), and 18–24 months (*n* = 620) from four independent studies in low socio‐economic populations in South Africa were pooled. A maximum‐likelihood factor model, with the principal‐factor method, was used to derive dietary (food) patterns. Associations between dietary pattern scores and nutrient intakes were determined using Kendall's Rank Correlations, with Bonferroni‐adjusted significance levels. For both 6–11 months and 12–17 months, the *formula milk/reverse breast milk* pattern was positively associated with energy and protein intake and mean adequacy ratio (MAR). The *family foods* pattern (6–11 months) and *rice and legume* pattern (12–17 months) were positively associated with plant protein, fibre, and PU fat; both for total intake and nutrient density of the complementary diet. These two patterns were also associated with the dietary diversity score (DDS; *r* = 0.2636 and *r* = 0.2024, respectively). The *rice* pattern (18–24 months) showed inverse associations for nutrient intakes and nutrient densities, probably because of its inverse association with fortified maize meal. The *more westernized* pattern (18–24 months) was positively associated with unfavourable nutrients, for example, saturated fat and cholesterol. These results highlight that underlying dietary patterns varied in terms of energy/nutrient composition, nutrient adequacy, nutrient densities of the complementary diet, and dietary diversity.

**Key Messages**

- The association of *formula milk/reverse breast milk* pattern scores and MAR suggests that breastfeeding children are more likely to consume a diet of lower nutrient adequacy.
- Associations of *formula milk/reverse breast milk* pattern scores and nutrient densities of the complementary diet suggest that breastfeeding children consume a complementary diet of lower nutrient density.
- The *more westernized* dietary pattern was associated with unfavourable nutrients such as saturated fat, cholesterol, and sugar, as well as certain micronutrients.
- Although associations of dietary pattern scores with dietary quality indicators could be explained by the foods with high factor loadings in most cases, this was not always the case.

## 1 INTRODUCTION

Optimal nutrition during infancy and early childhood is critical for child growth and development; and early feeding practices influence health later in life (Black et al., 2013). From age 6 to 24 months, infant feeding transitions progressively from predominantly breastfeeding (or milk feeds) to semisolid early infant foods to a variety of family foods. Dietary patterns that are established during the first 2 years of life (6–24 months of age) may track into midchildhood (Luque et al., 2018) and influence taste and food preference (Schwartz, Scholtens, Lalanne, Weenen, & Nicklaus, 2011). Dietary patterns of young children are affected and shaped by the caregiver (May & Dietz, 2010) and may be related to parental dietary patterns (Salles‐Costa, Barroso, Cabral, & de Castro, 2016). Distinct dietary patterns can be identified as early as age 6 months (Wen, Kong, Eiden, Sharma, & Xie, 2014), and consumption patterns may differ between breastfed and formula‐fed babies (Conn, Davies, Walker, & Moore, 2009; Noble & Emmett, 2006). A study involving 14‐month‐old children in the Netherlands showed that the distinct *Western‐like* dietary pattern and *health conscious* dietary pattern are already present at this young age (Kiefte‐de Jong et al., 2013).

Dietary patterns based on predefined dietary indices or derived from factor or cluster analyses examine the whole diet rather than individual foods and/or nutrients (Hu, 2002). In factor analyses, various arbitrary decisions are taken, including grouping of foods into food groups and the naming of the dietary pattern (Hu, 2002; Newby & Tucker, 2004). Dietary patterns derived through factor analysis may therefore not necessarily be comparable between studies or even age groups, and associations between dietary patterns and nutrient intakes are complex and may be difficult to interpret. For example, Smithers, Golley, et al. (2012) identified a *home‐made traditional* pattern for young children at age 6–8 and 15 months, but the association of this dietary pattern with the nutrient profile was inconsistent between the two age groups.

Dietary patterns have been shown to be associated with infant growth outcomes such as length‐for‐age z‐scores and BMI z‐scores (Wen et al., 2014). Understanding the energy and nutrient content and nutritional quality of specific dietary patterns therefore will provide valuable insight that may guide the development of appropriate nutrition messages/policies in terms of infant and young child feeding, particularly against the background of the triple burden of malnutrition in South Africa (stunting, overweight/obesity, and micronutrient deficiencies).

In vulnerable populations in South Africa, dietary intake in 6–24‐month‐old children can range from predominantly maize‐based to predominantly based on commercial infant foods (Faber, 2005; Faber, Laubscher, & Berti, 2016; Swanepoel et al., 2018). Pooling diverse dietary intake data would potentially provide a dataset with sufficient variation to determine the nutrient profile of a variety of dietary patterns. The aim of this study was to determine whether distinct dietary patterns are associated with energy/nutrient intakes and nutritional quality in 6–24‐month‐old South African children of low socio‐economic status.

## 2 METHODS

### 2.1 Study design

This study consisted of pooled single 24‐hr dietary recalls for 6–24 month‐old children previously collected in four independent studies. All study sites were of low socio‐economic status. In *Study 1* (Smuts et al., 2005) and *Study 2* (Faber, 2005; Faber, Kvalsvig, Lombard, & Benadé, 2005), dietary intake data were collected for children who participated in two independent randomized controlled trials (RCT) that were done in rural sites in KwaZulu‐Natal province. Study participants were recruited through an NGO‐driven community‐based health programme that operated through 12 health posts. Exclusion criteria were birth weight <2500 g and haemoglobin concentration < 80 g/L (both studies), premature birth (<37‐week gestation), and weight‐for‐length z‐score < –3 (*Study 1* only). Data collection was done at baseline (at age 6–12 months) and follow‐up (at age 12–18 months). In *Study 1*, additional data were collected 6 months after the completion of the RCT (at age 18–24 months). In *Study 3* (Smuts et al. 2019; Swanepoel et al., 2018), dietary intake data were collected for children who participated in an RCT that was done in a peri‐urban site in North West province. Study participants were recruited through primary health care facilities and house‐to‐house visits. Exclusion criteria included haemoglobin concentration *<*70 g/L, weight‐for‐length z‐score *<* –3, severe congenital abnormalities, infant known to be HIV positive, and infants known to be allergic/intolerant to peanuts, soy, cow's milk protein, or fish. Data were collected at baseline (at age 6 months), follow‐up (at age 12 months), and 6‐month post RCT (at age 18 months). In all three studies, dietary intake data were missing for children whose caregiver could not provide reliable information because the child was not in her permanent care during the 24‐hr recall period. *Study 4* (Faber et al., 2016) was a cross‐sectional dietary assessment study. Primary caregivers of randomly selected children, stratified per age category (6–11 months, 12–17 months, and 18–24 months), were recruited through house‐to‐house visits in two study sites, one rural and one urban, in KwaZulu‐Natal province.

Previously collected 24‐hr dietary recalls were recoded to ensure that coding and analysis were standardized across all dietary surveys and that all records were analysed with the same version of the food composition database. Estimated intake of breast milk was assumed according to age: 675 ml for partially breastfed infants at age 6–11 months, 615 ml at age 12–17 months and 550 ml at age 18–24 months (WHO, 1998). Exclusively breastfed or formula‐fed infants were excluded. The complementary diet was defined as all foods and beverages consumed, excluding breast milk and formula milk feeds. Formula milk powder mixed into porridge/infant cereal may affect the nutrient density of the complementary diet and was therefore coded separately from formula milk feeds, using dummy food codes. This allowed for formula milk powder mixed into food to be included when calculating the nutrient density of the complementary diet. Food intake was converted to energy and nutrients using Stata software and the 2017 South African Food Composition Database (SAFOODS, 2017), which includes an updated section on infant foods.

The final dataset included 3,336 24‐hr recalls. The data were stratified into three age groups, namely, 6–11 months (n = 1,585), 12–17 months (n = 1,131), and 18–24 months (n = 620).

Nutrient adequacy ratios (NAR) were calculated using age appropriate estimated average requirements (EAR) or, where there is no EAR, the Adequate Intakes (AI) of the Dietary Reference Intakes (DRIs) (Otten, Hellwig, & Meyers, 2006). To calculate the mean adequacy ratio (MAR), NARs > 1 were capped at 1, and the MAR was calculated as the average of the capped NARs.

Micronutrient densities per 100 kcal of the complementary diet (all foods excluding breast milk and other milk feeds) were calculated.

A dietary diversity score (DDS) was calculated for each child based on World Health Organisation/the United Nations Children's Fund (WHO/UNICEF) guidelines (WHO/UNICEF, 2010), although adapted to include breast milk and formula milk feeds (in the dairy group). The food groups used to calculate the DDS were (i) grains, roots, and tubers; (ii) legumes and nuts; (iii) milk and milk products; (iv) flesh foods; (v) eggs; (vi) vitamin A–rich fruits and vegetables; and (vii) other fruits and vegetables.

Individual food items were grouped into 36 foods (or groups) based on nutritional composition and similarity of foods. Energy contribution of the foods was calculated and expressed as a percentage of total energy intake. Daily energy intake values (expressed as percentage of total intake) for the 36 foods were used in a maximum‐likelihood factor model, with the principal factor method to derive estimates of dietary patterns; a varimax (orthogonal) rotation of the factor‐loading matrix was done to make interpretation easier. Derived components with an eigenvalue > 1.00 and also containing two or more original foods with loading factor ≥ 0.35 or ≤ –0.35 were retained in order to name the factors. Regression scoring was used for the set of retained factors. A higher factor score indicates higher adherence to the corresponding dietary pattern. These factor scores (continuous variables) were then used to determine associations of the dietary patterns with energy and nutrient intakes, MAR, nutrient densities of the complementary diet, and the DDS, using Kendall's rank correlations, with Bonferroni‐adjusted significance levels.

Data were further explored by stratifying the children according to dietary pattern tertiles (Ts) and then calculating the percentage consumers for the 36 foods within each tertile. Differences across the tertiles were determined using the Fisher exact test.

Ethical considerations

Ethical approval was not required as we used pooled data from previous studies.

## 3 RESULTS

### 3.1 Dietary patterns

In each of the three age categories, three dietary patterns were identified, which explained 38.6% (6–11 months), 37.8% (12–17 months), and 32.7% (18–24 months) of the variance (**Table** 1). The percentage of children who consumed foods during the recall period according to dietary pattern tertiles is given in **Tables** 2, 3, 4. For significant associations of these patterns with energy and nutrient intakes, MAR, DDS (**Table** 5), and nutrient densities of the complementary diet (**Table** 6), a correlation coefficient (*r*) of between –0.3 and 0.3 is considered weak, and associations with *r* ≤ –0.3 and *r* ≥ 0.3 will mostly be highlighted hereafter.

**Table 1:** Factor loadings for the three patterns in each of the age categories

	**6–11 Months**			**12–17 Months**			**18–24 Months**		
	**Factor 1**	**Factor 2**	**Factor 3**	**Factor 1**	**Factor 2**	**Factor 3**	**Factor 1**	**Factor 2**	**Factor 3**
Breast milk	**–0.948**	–0.143	–0.178	–0.301	–0.175	**–0.870**	**–0.523**	0.109	0.143
Formula milk^1^	**0.904**	–0.178	–0.181	–0.327	–0.254	**0.746**	–0.205	–0.197	–0.017
Milk	0.060	0.102	0.228	0.171	–0.141	0.112	0.030	**0.456**	–0.049
Dairy products, sweetened	0.009	–0.020	0.110	–0.008	–0.080	–0.028	–0.061	0.115	–0.005
Yoghurt	0.037	–0.074	0.083	–0.037	–0.009	0.031	–0.005	0.230	0.039
Jarred baby foods	0.108	–0.249	–0.108	–0.091	–0.194	0.001	–0.091	0.120	0.155
Infant cereal	0.108	**–0.594**	–0.059	–0.135	–0.244	0.065	–0.073	0.010	0.067
Baby porridge	–0.061	–0.198	–0.037	–0.044	–0.140	0.001	–0.083	0.100	0.174
Maize meal porridge^2^	0.027	**0.517**	**0.452**	0.264	0.169	0.023	0.050	0.042	**–0.914**
Cooked porridge, other^3^	0.046	–0.059	0.048	0.239	–0.211	–0.056	0.010	0.270	–0.138
Breakfast cereal	0.058	–0.035	0.110	0.263	–0.275	0.056	0.043	**0.413**	0.131
Bread^2^	0.001	0.256	0.012	0.037	0.319	0.038	0.329	–0.165	0.285
Bread spreads	–	–	–	–0.018	0.011	–0.031	–0.017	0.059	0.034
Rice	0.056	**0.577**	–0.042	–0.052	**0.580**	0.069	0.115	**–0.580**	**0.375**
Potato	0.057	0.226	–0.065	0.055	–0.104	0.083	0.123	–0.094	0.105
Legumes	0.022	**0.435**	–0.122	–0.109	**0.557**	0.010	–0.078	**–0.498**	–0.051
Eggs	–0.004	0.038	0.002	–0.055	–0.016	0.004	–0.032	0.051	0.025
Chicken	0.045	0.223	0.081	0.147	0.140	0.076	0.068	0.012	0.027
Meat	0.007	0.085	–0.009	–0.038	0.027	0.060	–0.048	0.042	0.133
Organ meat	–0.020	–0.006	–0.016	0.030	–0.110	–0.036	–0.056	0.105	–0.095
Fish	0.011	0.104	0.035	–0.023	0.037	–0.022	0.045	0.132	0.159
Vegetables	–0.011	0.194	0.020	0.066	0.063	–0.024	0.170	–0.211	–0.136
Fruit fresh	0.030	0.181	0.007	–0.068	0.152	0.032	–0.051	–0.090	0.169
Fruit dried	–0.047	0.025	–0.027	–0.022	0.023	–0.041	0.076	–0.050	0.008
Fruit juice	–0.018	–0.001	–0.019	–0.016	0.053	0.048	0.013	0.074	0.034
Fruit juice and dairy blend	0.044	0.061	0.016	–0.073	0.122	0.063	–0.176	–0.036	0.212
Sugar	0.014	0.040	**0.774**	**0.776**	0.064	0.075	**0.766**	0.013	–0.034
Sweets	0.013	0.030	0.010	–0.003	–0.052	0.006	0.120	0.125	0.055
Popcorn	–0.036	0.011	–0.021	0.128	–0.105	–0.044	0.053	0.122	–0.044
Salty snacks	–0.001	0.095	0.009	–0.040	0.064	0.017	0.075	0.086	0.108
Soup and sauces	0.010	0.122	0.022	0.072	0.007	–0.037	–0.051	0.048	0.019
Cake and cookies	–0.017	–0.040	0.018	0.034	–0.080	0.004	–0.080	0.145	0.042
Cold drinks	–0.021	0.049	–0.029	0.079	0.039	0.059	0.018	0.062	0.016
Tea, rooibos	0.065	–0.110	**0.491**	**0.702**	–0.178	0.089	**0.472**	0.212	–0.084
Tea	–0.012	0.058	0.272	0.161	**0.357**	0.054	**0.414**	–0.248	0.085
Margarine	–0.052	0.319	0.005	0.034	0.108	–0.042	0.215	–0.064	0.141
Eigenvalue	1.88	1.78	1.14	1.74	1.43	1.31	1.73	1.60	1.17
Variance explained	14.2%	13.8%	10.6%	14.0%	12.0%	11.8%	11.8%	10.8%	10.1%

1 Either as milk feeds or mixed with porridge/infant cereal.

2 Maize meal (used to make porridge) and wheat flour (used to make bread) are fortified as part of the National Food Fortification Programme (Department of Health, 2003).

3 Cooked porridge other than porridge made with maize meal.

Oil was excluded from the analysis, as oil used during food preparation was not always coded separately.

**Table 2:** Foods consumed; % consumers per dietary pattern; 6–11 months

	Factor 1				Factor 2				Factor 3			
	Formula milk/reverse breast milk				Family foods				Maize meal and sugar			
	T1	T2	T3	P‐value	T1	T2	T3	P‐value	T1	T2	T3	P‐value
Breast milk	100.0	99.8	11.9	**<0.001**	68.6	73.7	69.5	0.156	68.8	75.9	67.0	**0.003**
Formula milk^1^	7.6	34.1	86.2	**<0.001**	53.3	42.4	32.0	**<0.001**	56.1	38.8	32.8	**<0.001**
Baby jars	18.9	18.2	24.6	**0.019**	35.5	21.4	4.7	**<0.001**	34.6	14.6	12.5	**<0.001**
Baby porridge	18.9	7.8	10.2	**<0.001**	23.4	11.2	2.3	**<0.001**	15.9	12.9	8.1	**<0.001**
Infant cereal	34.4	41.3	43.6	**0.006**	74.9	35.6	8.7	**<0.001**	52.6	45.5	21.2	**<0.001**
Maize meal^2^	51.0	68.8	59.8	**<0.001**	11.7	75.0	93.0	**<0.001**	30.1	63.4	86.2	**<0.001**
Cooked porridge, other	0.9	2.5	3.0	**0.041**	2.8	2.1	1.5	0.354	0.9	2.8	2.7	**0.047**
Breakfast cereal	1.5	3.0	3.6	0.080	1.5	4.5	2.1	**0.007**	0.8	2.3	5.1	**<0.001**
Bread^2^	2.1	9.1	5.3	**<0.001**	0.4	1.7	14.4	**<0.001**	3.8	4.2	8.5	**<0.001**
Margarine	30.1	39.4	28.2	**<0.001**	6.2	39.2	52.3	**<0.001**	25.1	35.0	37.5	**<0.001**
Potato	15.5	26.9	22.5	**<0.001**	6.2	25.2	33.5	**<0.001**	23.4	20.6	20.8	0.474
Rice	6.8	31.4	19.1	**<0.001**	0.4	6.8	50.2	**<0.001**	19.1	20.6	17.6	0.453
Legumes	3.6	20.1	12.3	**<0.001**	0.4	3.8	31.8	**<0.001**	15.7	11.9	8.3	**<0.001**
Meat	0.8	3.0	2.1	**0.020**	0.0	2.3	3.6	**<0.001**	1.3	2.8	1.7	0.193
Chicken	0.4	5.5	4.9	**<0.001**	0.0	1.7	9.1	**<0.001**	1.1	3.8	5.9	**<0.001**
Organ meat	0.8	1.3	0.6	0.445	0.9	0.8	0.9	1.000	1.3	0.2	1.1	0.109
Fish	0.8	1.9	1.7	0.235	0.0	0.9	3.4	**<0.001**	0.8	1.1	2.5	0.060
Eggs	4.3	7.8	5.5	0.056	3.0	7.0	7.6	**0.002**	5.3	6.6	5.7	0.644
Milk	12.1	17.6	15.2	**0.040**	7.4	18.9	18.6	**<0.001**	5.5	15.5	23.9	**<0.001**
Vegetables	16.1	18.6	17.8	0.545	3.8	20.8	27.8	**<0.001**	13.8	19.1	19.5	**0.021**
Fruit fresh	7.6	19.7	14.2	**<0.001**	4.2	14.4	22.9	**<0.001**	8.9	17.4	15.2	**<0.001**
Fruit juice	1.1	0.9	0.6	0.708	1.1	0.6	0.9	0.708	1.1	0.9	0.6	0.708
Fruit juice, sweet	3.2	5.5	5.9	0.086	4.0	4.9	5.7	0.436	3.8	4.9	5.9	0.283
Tea, rooibos	3.0	3.4	7.8	**<0.001**	7.8	3.2	3.2	**<0.001**	0.4	4.2	9.7	**<0.001**
Tea	1.1	3.4	1.5	**0.026**	0.9	0.9	4.2	**<0.001**	0.6	0.9	4.5	**<0.001**
Sugar	39.7	49.1	46.6	**0.007**	30.4	48.5	56.4	**<0.001**	12.7	43.6	79.2	**<0.001**
Sweets	1.3	1.7	1.3	0.868	0.8	1.7	1.9	0.235	1.1	1.7	1.5	0.702
Cake and cookies	2.8	2.5	3.0	0.859	2.8	4.0	1.5	**0.045**	2.6	2.8	2.8	0.963
Cold drinks	2.6	4.9	2.5	0.060	1.5	3.8	4.7	**0.006**	3.0	3.0	4.0	0.639
Popcorn	1.1	0.9	0.2	0.176	0.6	1.3	0.4	0.235	0.9	1.1	0.2	0.115
Salty snacks	2.8	9.3	6.1	**<0.001**	2.6	7.2	8.3	**<0.001**	4.3	6.6	7.2	0.105
Soup and sauces	6.0	12.7	7.2	**<0.001**	2.6	7.0	16.3	**<0.001**	6.0	10.2	9.7	0.027
Yoghurt	8.3	8.9	12.7	**0.040**	9.8	14.2	5.9	**<0.001**	4.9	13.1	11.9	**<0.001**

1 Either as milk feeds or mixed with porridge/infant cereal.

2 Maize meal (used to make porridge) and wheat flour (used to make bread) are fortified as part of the National Food Fortification Programme (Department of Health, 2003).

P‐values based on Fischer exact test.

**Table 3:** Foods consumed; % consumers per dietary pattern; 12–17 months

	Factor 1				Factor 2				Factor 3			
	Tea and sugar				Rice and legumes				Formula milk/reverse breast milk			
	T1	T2	T3	P‐value	T1	T2	T3	P‐value	T1	T2	T3	P‐value
Breast milk	65.8	76.4	32.4	**<0.001**	59.2	64.7	50.7	**<0.001**	100.0	70.3	4.2	**<0.001**
Formula milk^1^	44.6	19.1	9.8	**<0.001**	36.3	24.4	12.7	**<0.001**	4.2	11.1	58.1	**<0.001**
Baby jars	7.4	4.2	2.7	**0.010**	11.7	2.1	0.5	**<0.001**	5.6	3.7	5.0	0.459
Baby porridge	10.1	5.6	6.4	**0.048**	14.1	5.3	2.7	**<0.001**	9.0	5.0	8.0	0.092
Infant cereal	17.5	6.1	3.7	**<0.001**	20.4	4.0	2.9	**<0.001**	7.4	8.8	11.1	0.215
Maize meal^2^	82.2	92.3	92.3	**<0.001**	78.2	94.7	93.9	**<0.001**	86.2	92.8	87.8	**0.008**
Cooked porridge, other	5.3	11.4	17.2	**<0.001**	22.5	9.8	1.6	**<0.001**	14.1	11.1	8.8	0.072
Breakfast cereal	4.0	7.4	18.0	**<0.001**	22.3	5.3	1.9	**<0.001**	8.0	9.8	11.7	0.242
Bread^2^	17.0	18.6	20.2	0.557	5.8	15.9	34.0	**<0.001**	13.0	23.6	19.1	**0.001**
Margarine	37.7	42.7	36.1	0.151	28.4	43.8	44.3	**<0.001**	40.3	39.5	36.6	0.554
Potato	24.7	31.3	35.0	**0.007**	38.2	33.2	19.6	**<0.001**	24.7	30.2	36.1	**0.003**
Rice	57.0	53.6	40.1	**<0.001**	15.1	53.6	82.0	**<0.001**	48.5	48.3	53.8	0.229
Legumes	32.9	25.2	15.4	**<0.001**	0.5	16.2	56.8	**<0.001**	24.1	26.3	23.1	0.587
Meat	13.0	13.0	7.4	**0.017**	9.5	13.0	10.9	0.328	5.8	13.3	14.3	**<0.001**
Chicken	13.8	23.9	26.8	**<0.001**	14.1	24.4	26.0	**<0.001**	14.9	24.9	24.7	**<0.001**
Organ meat	2.4	2.7	3.4	0.729	6.6	1.9	0.0	**<0.001**	2.4	3.4	2.7	0.729
Fish	5.3	6.1	6.1	0.877	2.7	8.8	6.1	**0.001**	4.5	7.2	5.8	0.323
Eggs	11.7	7.2	5.8	**0.011**	8.2	9.8	6.6	0.293	6.6	8.8	9.3	0.370
Milk	21.8	38.7	53.8	**<0.001**	48.3	39.8	26.3	**<0.001**	32.1	41.6	40.6	**0.012**
Vegetables	26.0	37.9	39.5	**<0.001**	25.7	42.7	35.0	**<0.001**	35.0	35.3	33.2	0.806
Fruit fresh	33.4	32.1	23.6	**0.005**	17.2	32.4	39.5	**<0.001**	22.8	34.5	31.8	**0.001**
Fruit juice	2.1	1.3	1.6	0.773	1.3	1.3	2.4	0.462	1.1	0.5	3.4	**0.007**
Fruit juice, sweet	17.8	14.1	8.5	**0.001**	10.3	12.2	17.8	**0.009**	8.5	15.9	15.9	**0.002**
Rooibos	2.9	5.0	30.2	**<0.001**	22.3	10.9	5.0	**<0.001**	7.7	14.6	15.9	**0.001**
Tea	5.0	10.6	19.9	**<0.001**	1.3	6.9	27.3	**<0.001**	6.6	17.5	11.4	**<0.001**
Sugar	50.9	79.6	89.9	**<0.001**	63.4	75.6	81.4	**<0.001**	67.4	78.0	75.1	**0.003**
Sweets	3.4	5.6	4.2	0.394	6.4	4.2	2.7	**0.048**	2.9	6.4	4.0	**0.073**
Cake and cookies	5.8	8.2	11.7	**0.018**	13.8	9.0	2.9	**<0.001**	5.8	12.7	7.2	**0.002**
Cold drinks	7.7	14.1	17.0	**<0.001**	14.6	14.1	10.1	0.129	9.5	17.0	12.2	**0.009**
Popcorn	1.9	4.2	7.7	**0.001**	9.0	3.4	1.3	**<0.001**	4.8	5.6	3.4	0.390
Salty snacks	21.8	20.7	18.0	0.417	20.2	21.5	18.8	0.680	16.4	23.9	20.2	**0.041**
Soup and sauces	17.5	23.6	21.2	0.117	23.1	21.8	17.5	0.138	19.4	21.2	21.8	0.708
Yoghurt	16.2	13.8	12.7	0.394	16.2	13.5	13.0	0.423	9.8	16.7	16.2	**0.009**

1 Either as milk feeds or mixed with porridge/infant cereal.

2 Maize meal (used to make porridge) and wheat flour (used to make bread) are fortified as part of the National Food Fortification Programme (Department of Health, 2003).

P‐values based on Fischer exact test.

**Table 4:** Foods consumed; % consumers per dietary pattern; 18 – 24 months

	Factor 1				Factor 2				Factor 3			
	Tea and sugar				More westernized				Rice			
	T1	T2	T3	p‐value	T1	T2	T3	p‐value	T1	T2	T3	p‐value
Breast milk	66.7	6.3	1.5	**<0.001**	10.6	33.3	30.6	**<0.001**	18.4	27.1	29.1	**0.024**
Formula milk^1^	19.8	10.1	2.9	**<0.001**	21.7	8.2	2.9	**<0.001**	8.7	19.8	4.4	**<0.001**
Baby jars	1.9	0.0	0.5	0.093	0.0	0.0	2.4	**0.004**	0.0	0.5	1.9	**0.052**
Baby porridge	2.9	0.0	1.0	**0.024**	0.5	0.5	2.9	**0.040**	0.0	1.0	2.9	**0.015**
Infant cereal	4.3	1.4	0.0	**0.004**	1.9	1.4	2.4	0.721	0.5	2.4	2.9	0.131
Maize meal^2^	92.8	93.7	88.3	0.119	91.8	93.2	89.8	0.452	100.0	99.0	75.7	**<0.001**
Cooked porridge, other	12.6	15.0	7.8	0.061	0.5	6.8	28.2	**<0.001**	17.4	11.1	6.8	**0.004**
Breakfast cereal	11.1	11.6	6.3	0.120	0.5	1.4	27.2	**<0.001**	6.8	8.2	14.1	**0.033**
Bread^2^	17.4	30.0	50.5	**<0.001**	38.2	37.2	22.3	**<0.001**	17.4	33.8	46.6	**<0.001**
Margarine	34.8	33.3	45.6	**0.020**	41.1	45.4	27.2	**<0.001**	26.1	40.6	47.1	**<0.001**
Potato	30.9	35.3	34.5	0.621	31.4	34.3	35.0	0.725	30.0	35.7	35.0	0.399
Rice	61.4	52.7	64.1	0.051	88.4	63.8	25.7	**<0.001**	32.9	67.1	78.2	**<0.001**
Legumes	30.4	30.9	21.8	0.066	57.0	20.3	5.8	**<0.001**	22.7	35.3	25.2	**0.011**
Meat	21.3	24.6	13.1	**0.008**	15.5	22.2	21.4	0.163	15.9	17.4	25.7	**0.029**
Chicken	26.6	32.4	29.1	0.426	20.8	33.8	33.5	**0.003**	30.9	28.5	28.6	0.841
Organ meat	4.3	1.9	1.9	0.263	0.0	2.4	5.8	**<0.001**	4.8	2.4	1.0	0.061
Fish	7.7	12.1	6.8	0.146	4.3	11.1	11.2	**0.015**	5.3	6.8	14.6	**0.003**
Eggs	10.6	9.7	7.8	0.627	5.3	10.6	12.1	**0.033**	6.8	10.1	11.2	0.261
Milk	41.5	54.1	36.4	**0.001**	18.4	37.7	76.2	**<0.001**	46.9	43.0	42.2	0.602
Vegetables	34.8	34.3	51.0	**<0.001**	43.5	44.4	32.0	**0.016**	44.4	43.0	32.5	**0.025**
Fruit fresh	35.7	42.5	31.6	0.068	38.2	42.5	29.1	**0.015**	25.1	39.6	45.1	**<0.001**
Fruit juice	0.5	1.9	2.9	0.152	0.0	1.9	3.4	**0.013**	1.9	1.9	1.5	1.000
Fruit juice, sweet	24.2	19.8	5.8	**<0.001**	15.5	20.8	13.6	0.129	5.3	19.3	25.2	**<0.001**
Rooibos	6.8	14.0	34.0	**<0.001**	8.7	13.0	33.0	**<0.001**	24.6	14.5	15.5	**0.016**
Tea	12.1	27.5	46.1	**<0.001**	39.6	30.4	15.5	**<0.001**	24.6	27.1	34.0	0.097
Sugar	61.8	81.2	97.6	**<0.001**	83.6	77.8	79.1	0.293	84.1	79.2	77.2	0.192
Sweets	5.8	8.2	12.1	0.073	3.9	7.2	15.0	**<0.001**	10.1	6.8	9.2	0.429
Cake and cookies	15.5	11.1	7.8	**0.049**	4.8	10.1	19.4	**<0.001**	10.6	11.6	12.1	0.878
Cold drinks	20.8	24.2	19.9	0.555	14.5	20.3	30.1	**0.001**	25.1	22.2	17.5	0.158
Popcorn	3.9	4.3	5.3	0.739	0.5	3.4	9.7	**<0.001**	7.2	3.4	2.9	0.095
Salty snacks	21.3	31.4	29.1	**0.049**	15.9	30.0	35.9	**<0.001**	28.5	22.7	30.6	0.172
Soup and sauces	26.6	23.7	15.0	**0.011**	12.1	26.6	26.7	**<0.001**	19.8	22.7	22.8	0.710
Yoghurt	13.5	22.2	13.6	**0.027**	6.8	14.0	28.6	**<0.001**	15.9	19.3	14.1	0.349

1 Either as milk feeds or mixed with porridge/infant cereal.

2 Maize meal (used to make porridge) and wheat flour (used to make bread) are fortified as part of the National Food Fortification Programme (Department of Health, 2003).

P‐values based on Fischer exact test.

**Table 5:** Correlations of energy and nutrient intakes with dietary patterns; Kendall's Rank Correlations, with Bonferroni‐adjusted significance levels (*P < 0.05)

	6–11 Months			12–17 Months			18–24 Months		
	Factor 1	Factor 2	Factor 3	Factor 1	Factor 2	Factor 3	Factor 1	Factor 2	Factor 3
	Formula milk/reverse breast milk	Family foods	Maize meal and sugar	Tea and sugar	Rice and legumes	Formula milk/reverse breast milk	Tea and sugar	More westernized	Rice
kJ	0.3380*	0.2570*	0.1605*	0.0365	0.1423*	0.2633*	–0.0859	0.1054	–0.0759
Protein	0.4177*	0.1925*	0.1132*	0.0658	0.1169*	0.3125*	–0.0579	0.1380*	–0.0149
Plant protein	0.2009*	0.5810*	0.2365*	0.1920*	0.4516*	0.2092*	0.1373*	–0.1706*	–0.1432*
Animal protein	0.2856*	0.1824*	0.0807*	0.0871*	–0.0501	0.2423*	–0.0456	0.2448*	0.0506
Fat	0.1297*	0.2403*	0.0394	–0.1480*	0.0262	0.0475	–0.2652*	0.1458*	0.1322*
Saturated fat	–0.2634*	0.1290*	0.1062*	–0.0262	–0.0571	–0.2328*	–0.2937*	0.3530*	0.1152*
MU fat	–0.2766*	0.2396*	0.1175*	–0.0219	0.0607	–0.2428*	–0.2175*	0.2495*	0.1370*
PU fat	–0.0729*	0.4522*	0.1487*	0.0711	0.3669*	0.0373	0.0803	–0.1142*	0.0808
Cholesterol	–0.3755*	0.0486	0.0586	–0.0281	–0.0780	–0.2673*	–0.2524*	0.3108*	0.1121*
CHO	0.3700*	0.2180*	0.2080*	0.1312*	0.1813*	0.2933*	0.0304	0.0424	–0.1996*
Sugar	–0.3515*	–0.0106	0.1704*	0.0904*	–0.023	–0.2857*	–0.1171*	0.2554*	0.0929
Fibre	0.2169*	0.4969*	0.2164*	0.1485*	0.3822*	0.1906*	0.0747	–0.1319*	–0.1498*
Calcium	0.3670*	–0.1862*	–0.0367	–0.1310*	–0.2131*	0.2600*	–0.2688*	0.2714*	0.0271
Iron	0.4764*	–0.1673*	–0.0200	–0.0025	–0.0254	0.4899*	–0.0111	0.0359	–0.1331*
Magnesium	0.3830*	0.4231*	0.2085*	0.1953*	0.1982*	0.3095*	0.0070	0.0969	–0.3147*
Phosphorous	0.4707*	0.1347*	0.1136*	0.0908*	0.0428	0.3816*	–0.0697	0.1969*	–0.1353*
Potassium	0.4075*	0.1564*	0.0119	–0.0214	0.0828*	0.3287*	–0.1228*	0.0317	0.0632
Zinc	0.5885*	0.1220*	0.0722*	0.0119	0.0505	0.4378*	–0.0425	0.0508	–0.2010*
Copper	0.2738*	0.2766*	–0.0216	–0.1078*	0.0875*	0.1371*	–0.1523*	–0.0097	0.0301
Vitamin A	0.2350*	–0.1361*	–0.0727*	–0.1327*	–0.2052*	0.0922*	–0.2869*	0.1790*	–0.2110*
Thiamine	0.5723*	0.0873*	0.0697*	0.0429	0.0794	0.4332*	–0.0282	0.0317	–0.3044*
Riboflavin	0.5223*	–0.0567	0.0079	–0.039	–0.1798*	0.3541*	–0.0934	0.3101*	–0.0477
Niacin	0.4626*	0.1540*	0.0683*	0.1014*	0.1617*	0.3503*	0.0628	0.0079	–0.0261
Vitamin B6	0.4347*	0.3224*	0.1592*	0.0928*	0.2850*	0.3246*	0.1777*	–0.0494	–0.0861
Folate	0.3725*	0.1925*	0.2205*	0.1227*	0.1151*	0.2947*	0.0217	0.0652	–0.4069*
Vitamin B12	0.4284*	–0.0942*	–0.041	–0.0836*	–0.2256*	0.2473*	–0.1584*	0.3111*	0.0585
Pantothenic acid	0.5141*	–0.0144	–0.0803*	–0.1179*	–0.0910*	0.2962*	‐0.2209*	0.1265*	0.0023
Biotin	0.5392*	0.0509	0.0341	0.0124	–0.0735	0.4476*	0.0000	0.1211*	–0.1560*
Vitamin C	0.3332*	–0.2043*	–0.1638*	–0.3361*	–0.1205*	0.1694*	–0.2556*	–0.1028	0.1665*
Vitamin D	0.5269*	–0.1195*	–0.0969*	–0.2318*	–0.1745*	0.2886*	–0.1839*	0.1020	0.1273*
Vitamin E	0.5512*	0.0190	–0.0545	–0.1012*	0.0668	0.4673*	0.0727	‐0.1611*	0.0572
MAR	0.5769*	0.0958*	0.0543	–0.0517	–0.1109	0.3598*	–0.2534*	0.2503*	–0.0376
DDS	0.0919*	0.2636*	–0.0036	–0.0610	0.2024*	0.0569	–0.1558*	0.0397	0.1270*

DDS, dietary diversity score; MAR, mean adequacy ratio.

**Table 6:** Correlations of nutrient densities (per 100 kcal) of the complementary diet with dietary patterns; Kendall's rank correlations, with Bonferroni‐adjusted significance levels (*P < 0.05)

	6–11 Months			12–17 Months			18–24 Months		
	Factor 1	Factor 2	Factor 3	Factor 1	Factor 2	Factor 3	Factor 1	Factor 2	Factor 3
	Formula milk/reverse breast milk	Family foods	Maize meal and sugar	Tea and sugar	Rice and legumes	Formula milk/reverse breast milk	Tea and sugar	More westernized	Rice
Protein	0.2489*	0.1807*	0.2234*	0.2872*	0.1810*	0.2411*	0.1639*	0.1005	0.0269
Plant protein	0.1633*	0.5916*	0.2459*	0.2463*	0.5082*	0.1488*	0.2738*	–0.3217*	–0.1212*
Animal protein	0.0868*	0.2113*	0.1354*	0.1813*	–0.0363	0.1263*	0.0146	0.2671*	0.0727
Fat	0.2168*	0.3331*	0.2247*	0.2699*	0.2052*	0.2222*	0.2925*	0.1174*	0.1679*
Saturated fat	0.1092*	0.2446*	0.2356*	0.2393*	0.0068	0.1607*	0.1335*	0.3686*	0.1159*
MU fat	0.1017*	0.3897*	0.2411*	0.2370*	0.1626*	0.1632*	0.2279*	0.1737*	0.1627*
PU fat	0.1043*	0.5739*	0.2218*	0.1880*	0.4225*	0.1323*	0.2820*	–0.2129*	0.1075*
Cholesterol	0.0506	0.1246*	0.1362*	0.1531*	–0.0487	0.1196*	0.0031	0.3006*	0.0945
CHO	0.2841*	0.1955*	0.3680*	0.4676*	0.2527*	0.2790*	0.4020*	–0.0831	–0.2121*
Sugar	0.0673*	–0.0458	0.2726*	0.3183*	–0.0121	0.1383*	0.3185*	0.1259*	0.0802
Fibre	0.1757*	0.4960*	0.2104*	0.1800*	0.4016*	0.1307*	0.1591*	–0.2433*	–0.1209*
Calcium	0.1307*	–0.2742*	0.0337	0.1374*	–0.1064*	0.1561*	0.0579	0.2812*	0.0388
Iron	0.1493*	–0.2476*	0.0222	0.2248*	**0.0271**	0.2293*	0.1833*	0.0625	–0.1334*
Magnesium	0.1892*	0.4835*	0.3183*	0.4322*	0.2494*	0.2092*	0.1929*	0.0412	–0.3854*
Phosphorous	0.2306*	0.1200*	0.2097*	0.3229*	0.1094*	0.2382*	0.1485*	0.1895*	–0.1355*
Potassium	0.2168*	0.1161*	0.0704*	0.2232*	0.1482*	0.2565*	0.1797*	–0.0334	0.1083*
Zinc	0.2200*	0.1745*	0.2804*	0.3564*	0.1668*	0.2226*	0.2138*	0.0511	–0.2402*
Copper	0.2327*	0.3708*	0.1168*	0.2979*	0.2563*	0.2146*	0.3312*	–0.1043	0.0502
Vitamin A	0.1011*	–0.1906*	0.0373	0.2073*	–0.1230*	0.0982*	0.1094*	0.2001*	–0.3300*
Thiamine	0.2313*	0.0857*	0.2149*	0.3182*	0.1591*	0.2152*	0.1731*	0.0084	–0.3903*
Riboflavin	0.1523*	–0.1100*	0.1443*	0.2163*	–0.1160*	0.1756*	0.1114*	0.3592*	–0.0528
Niacin	0.1848*	0.1934*	0.1800*	0.3001*	0.2273*	0.2090*	0.2812*	‐0.0509	0.0344
Vitamin B6	0.1824*	0.3749*	0.2768*	0.2430*	0.3620*	0.1986*	0.3331*	‐0.1170*	–0.0755
Folate	0.0884*	0.2165*	0.3661*	0.2688*	0.1435*	0.0988*	0.1306*	0.0391	–0.4533*
Vitamin B12	0.1439*	–0.1205*	0.0601	0.1027*	–0.1631*	0.1103*	‐0.0095	0.3645*	0.0665
Pantothenic acid	0.2585*	0.0018	0.0774*	0.2117*	0.0360	0.2289*	0.1183*	0.1284*	0.0200
Biotin	0.1624*	0.0957*	0.1472*	0.2193*	–0.0181	0.1511*	0.1551*	0.1806*	–0.1416*
Vitamin C	0.1189*	–0.3136*	–0.1152*	–0.0786	–0.0819*	0.1427*	0.0080	–0.0863	0.1682*
Vitamin D	0.1612*	–0.1893*	–0.0336	0.0011	–0.0674	0.0940*	0.0382	0.1842*	0.1576*
Vitamin E	0.1653*	0.0528	0.0396	0.0948*	0.1927*	0.1483*	0.2265*	–0.1596*	0.1184*

#### 3.1.1 Age 6–12 months:

Factor 1, named the ‘formula milk/reverse breast milk' pattern, had a very high positive loading for formula milk and a very high negative loading for breast milk (**Table** 1), indicating an inverse association between formula milk and breast milk. In terms of pattern score tertiles (**Table** 2), 7.6% of children consumed formula milk in T1 versus 86.2% in T3. The opposite was observed for breast milk, with all children in T1 receiving breast milk, versus 11.9% in T3. The ‘formula milk/reverse breast milk' pattern was positively associated with energy, protein and most micronutrients, and ultimately MAR (**Table** 5), as well as with the nutrient density of the complementary diet for various nutrients (**Table** 6), although these associations were weak (*r* > –0.3 and *r* < 0.3).

Factor 2, named the ‘family foods' pattern, had high positive loadings for maize meal, rice, and legumes and a high negative loading for infant cereal. The ‘family foods' pattern was inversely associated with all commercial infant products and positively associated with several family foods (**Table** 2). In terms of nutrients, the ‘family food' pattern was positively associated with plant protein, fibre, and PU fat; both for total intake (**Table** 5) and the nutrient density of the complementary diet (**Table** 6). This pattern was positively associated (*r* ≥ 0.3) with magnesium and vitamin B6, both for total intake and nutrient density of the complementary diet, and inversely associated with the nutrient density of the complementary diet for vitamin C and, to a lesser extent, calcium (*r* = –0.2742) and iron (*r* = –0.2476).

Factor 3, named the ‘maize meal and sugar' pattern, had a high loading for maize meal. The ‘maize meal and sugar' pattern was inversely associated with all commercial infant products (**Table** 2). This dietary pattern showed statistically significant correlations with a few nutrient intakes (**Table** 5) and nutrient densities for various micronutrients (**Table** 6), but most of these correlations were weak (*r* > –0.3 and *r* < 0.3), except for the nutrient densities for carbohydrates, magnesium, and folate (*r* ≥ 0.3).

#### 3.1.2 Age 12–17 months:

Factor 1, named the ‘tea and sugar' pattern, had high loadings for sugar and rooibos tea. This pattern was not associated with energy and nutrient intakes, or MAR (**Table** 5), but it was associated with the nutrient density of the complementary diet for several micronutrients (**Table** 6).

Factor 2, named the ‘rice and legumes' pattern, had high loading for rice, legumes, and tea (**Table** 1). This pattern was positively associated with plant protein, fibre, and PU fat, both for total intake (**Table** 5) and the nutrient density of the complementary diet (**Table** 6 **)**.

Factor 3, named the ‘formula milk/reverse breast milk' pattern, had a high positive loading for formula milk and a high negative loading for breast milk (**Table** 1). In terms of pattern score tertiles (**Table** 3), 4.2% of children consumed formula milk in T1 versus 58.1% in T3. The opposite was observed for breast milk, with all children in T1 receiving breast milk versus 4.2% in T3. The ‘formula milk/reverse breast milk' pattern was positively associated with energy, protein and most micronutrients, and ultimately MAR (**Table** 5). This pattern was also positively associated with the nutrient density of the complementary diet for various nutrients (**Table** 6), although these associations were weak (*r* > –0.3 and *r* < 0.3).

#### 3.1.3 Age 18–24 months:

Factor 1, named the ‘tea and sugar' pattern had a high loading for tea, rooibos tea, and sugar and a high negative loading for breast milk (**Table** 1). This dietary pattern showed several statistically significant inverse correlations with nutrient intakes, but these correlations were weak (*r* > –0.3 and *r* < 0.3).

Factor 2, named the ‘more westernized' pattern had high loadings for breakfast cereal and milk and high negative loadings for rice and legumes (**Table** 1), therefore indicating a less traditional but more westernized diet. This pattern was associated with a higher percentage consumers of unhealthy food items such as sweets, cake and cookies, cold drinks, and salty snacks (**Table** 4). In terms of nutrients (**Table** 6), this pattern was associated with saturated fat, cholesterol, and riboflavin intakes.

Factor 3, named the ‘rice' pattern, had a high loading for rice and a high negative loading for maize meal (**Table** 1). This pattern showed several statistically significant inverse correlations but *r* ≥ 0.3 for only magnesium, thiamine, and folate.

#### 3.1.4 Dietary diversity

The ‘family foods' pattern (6–11 months; *r* = 0.2636) and the ‘rice and legume' pattern (12–17 months; *r* = 0.2024) were associated with the DDS, although these associations were weak (*r* > –0.3 and *r* < 0.3).

## 4 DISCUSSION

In this paper, we describe dietary patterns for 6–24‐month‐old children, using a large dataset of pooled single 24‐hr recalls previously collected in four independent studies done in areas of low socio‐economic status. Distinct dietary patterns were identified, and the results highlight that the underlying dietary patterns vary in terms of energy and nutrient composition, MAR, nutrient densities of the complementary diet, and DDS.

For 6–11 months and 12–17 months, a dietary pattern with a high positive loading for formula milk and a high negative loading for breast milk was identified. A similar dietary pattern with strong inverse association between formula milk and breast milk was reported for 6‐month‐old infants by Wen et al. (2014). In our study, the ‘formula milk/reverse breast milk' pattern was positively associated with energy, protein and most micronutrients, and ultimately MAR. Smithers, Brazionis, et al. (2012) also reported a dietary pattern with high loadings for breast milk and formula milk but in opposite directions. They showed that the ‘breastfeeding' pattern, which had a high negative loading for formula milk, was associated with lower energy‐adjusted micronutrient intakes for various key micronutrients, for example, calcium, iron, and zinc (Smithers, Golley, et al., 2012). Results of both our study and the study by Smithers, Golley et al. (2012) suggest that breastfeeding children are more likely to consume a diet of lower micronutrient content, which was reflected by the positive association of the ‘formula milk/reverse breast milk' pattern with the MAR in our study. Smithers, Golley, et al. (2012) however cautioned the interpretation of these results because of the limitations in estimating breast milk intake. Despite these limitations, the associations observed of the ‘formula milk/reverse breast milk' pattern with the nutrient density of the complementary diet for various nutrients, although weak (*r* > –0.3 and *r* < 0.3), suggest that breastfeeding children consume a complementary diet of lower nutrient density. We can only speculate on why this is the case. A study in South Africa reported that mothers who were breastfeeding were more likely to be unemployed compared with mothers who formula fed (Nieuwoudt, Manderson, & Norris, 2018) suggesting that income may be a factor. Nonetheless, these results suggest that a stronger focus is needed on the nutritional quality of the complementary foods for breastfeeding babies.

The ‘family foods' pattern (age 6–11 months) was positively associated with plant protein and fibre for total intake as well as the nutrient density of the complementary diet, indicating a mostly plant‐based diet. The association with PU fat can most probably be ascribed to oil used when preparing legumes. This pattern was positively associated with maize meal and inversely associated with infant cereals, both of which are fortified with various micronutrients. This may explain the inconsistent associations of this pattern with nutrient intakes and nutrient densities. While this pattern was positively associated (*r* ≥ 0.3) with magnesium and vitamin B6, both for total intake and nutrient density of the complementary diet, it was inversely associated with the nutrient density of the complementary diet for calcium (*r* = –0.2742) and iron (*r* = –0.2476). These inverse associations can probably be ascribed to the fact that maize meal is not fortified with calcium (Department of Health, 2003) and because the iron content of fortified infant cereals is considerably higher than the iron content in fortified maize meal (SAFOODS, 2017).

Besides for the ‘family foods' pattern, maize meal had a high loading for the ‘maize meal and sugar pattern' and the ‘rice pattern' (negative loading). Magnesium and folate were positively associated with the ‘maize meal and sugar' pattern and inversely associated with the ‘rice' pattern. Folate is one of the fortification nutrients used in the fortification of maize meal as part of the National Food Fortification Programme (Department of Health, 2003). Fortification of maize meal and the inverse associations of the ‘rice' pattern with maize meal may also explain the inverse association of this pattern with nutrient densities of the complementary diet for various nutrients, particularly zinc, vitamin A, thiamine, and folate (all of these are fortification nutrients).

Results further showed that a ‘more westernized' dietary pattern was associated with unfavourable nutrients such as saturated fat, cholesterol, and sugar, and consumption of sweets, cake and cookies, cold drinks, and salty snacks. At the same time, this dietary pattern (which had a high loading for milk) was positively associated with calcium, riboflavin, and vitamin B12 (nutrients found in milk). This highlights that a specific dietary pattern may be associated with nutrients that are protective and nutrients that should be consumed in moderation.

Both sugar and tea (either tea or rooibos tea) had high loadings in three factors. For the ‘tea and sugar' pattern for age 18–24 months, the high loadings for both food items can probably be explained by tea taken with sugar. For the two younger age groups, however, the percentage of children who consumed sugar across the tertiles for the ‘maize meal and sugar pattern' (6–11 months) and the ‘tea and sugar' pattern (12–17 months) were substantially higher than the percentage of children who consumed tea and/or rooibos tea. The inverse association of the ‘tea and sugar pattern' with both breast milk and formula milk (in both age groups) probably reflect mothers substituting breast milk/formula milk with tea as children grow older. In line with WHO/UNICEF (2003) guidelines, the proposed South African paediatric food‐based dietary guidelines recommend continued breastfeeding to 2 years and beyond, while tea, coffee, and sugary drinks should be avoided (Vorster, Badham, & Venter, 2013). Continued breastfeeding during the second year of life is however low in South Africa; according to the 2016 SADHS, 46.7% of 12–17‐month‐old children and 18.5% of 18–23‐month‐old children were breastfeeding (National Department of Health et al., 2019). The ‘tea and sugar' pattern for both age groups (12–17 months and 18–24 months) was associated with the nutrient density of the complementary diet for several micronutrients. As neither tea, rooibos tea, nor sugar provide micronutrients, the association with the micronutrient density of the complementary diet cannot be ascribed to the foods with high loadings in these patterns.

The positive association of both the ‘formula milk/reverse breast milk' pattern and the ‘tea and sugar' pattern with the nutrient density of the complementary diet may suggest a perception that as long as children are being breastfed, the quality of the complementary diet is not of that high importance. Although this is pure speculation, it warrants further investigation.

Dietary patterns identified in our study are based on a single 24‐hr recall, which has several inherent limitations (Murphy, Guenther, & Kretsch, 2006). Studies reporting dietary patterns in children of similar age group used either single 24‐hr recall (Gatica, Barros, Madruga, Matijasevich, & Santos, 2012; Melaku et al., 2018) or a food frequency questionnaire (Betoko et al., 2013; Smithers, Golley, et al., 2012; Wen et al., 2014). Robinson et al. (2007) reported that principle component analysis yielded similar patterns when a 24‐hr dietary recall was used compared with a food frequency questionnaire in 6‐month‐old infants. It should further be noted that we determined associations of dietary patterns with indicators of dietary quality; we did not assess associations of dietary patterns with health outcomes, in which case the use of single 24‐hr recall data would have been problematic.

The time interval between the original studies and the time lapse since data collection should have little impact on the results of this study, as the focus of this paper is on associations of dietary (food) patterns with indicators of dietary quality, which are not time‐bounded or affected by any external factors. As the SAFOODS is continuously being updated, we reanalysed all 24‐hr recalls using the most current version of the database (SAFOODS, 2017). Possible bias due to different versions of the database being used to convert food intake data to nutrient intake data was therefore avoided.

In conclusion, dietary patterns varied in terms of energy and nutrient composition, MAR, nutrients densities of the complementary diet, and DDS. Interpretation of the associations between pattern scores and indicators of dietary quality is complex, for various reasons. Firstly, although in most cases the associations could be explained by the foods with high loadings, this was not always the case. Secondly, some dietary patterns had both positive and negative associations with key micronutrients, particularly in the younger age group, probably because both infant cereals and maize meal are fortified. Lastly, the associations of the ‘formula milk/reverse breast milk' pattern score with various indicators of dietary quality need further attention, as these associations imply poorer dietary quality for breastfeeding babies.

## CONFLICT OF INTEREST

The authors declare that they have no conflicts of interest.

## CONTRIBUTIONS

The authors' responsibilities were as follows: M.F. conceptualized the study, wrote the first draft, and was responsible for collecting dietary intake data for all the original studies; M.R. contributed to dietary coding and was involved in collecting dietary data in one of the original studies and writing of the manuscript; R.L. did the data analyses; and C.M.S. was the principle investigator for two of the original studies. All authors read and approved the final manuscript.

## FUNDING INFORMATION

The study was funded by the South African Sugar Association (Project 247).
